# Association of vitamin d with glycemic control in Saudi patients with type 2 diabetes: A retrospective chart review study in an emerging university hospital

**DOI:** 10.1002/jcla.23048

**Published:** 2019-09-30

**Authors:** Khaled K. Al Dossari, Gulfam Ahmad, Abdulrahman Aljowair, Naif Alqahtani, Mohammed Bin Shibrayn, Mohammed Alshathri, Dahfer Alshehri, Suhair Akhlaq, Faisal Bin Hejab, Abdulelah Alqahtani, Hira Abdul Razzak

**Affiliations:** ^1^ College of Medicine Prince Sattam Bin Abdulaziz University Al‐Kharj Saudi Arabia; ^2^ Diabetes Centre Royal Prince Alfred Hospital Sydney New South Wales Australia; ^3^ Department of Physiology University of Health Sciences Lahore Pakistan; ^4^ Discipline of Pathology School of Medical Sciences Sydney medical School Sydney University Camperdown New South Wales Australia; ^5^ Ministry of Health and Prevention Dubai UAE

**Keywords:** diabetes, glycemic control, HbA1c, Saudi Arabia, vitamin D

## Abstract

**Background:**

Vitamin D (mainly 25‐hydroxyvitamin D, 25[OH]D) has stimulated increasing interest in Saudi Arabia over the current years due to its association with several different chronic diseases such as diabetes. This study aims to ascertain whether the vitamin D level has any influence on glycemic control in Saudi patients with type 2 diabetes (T2DM).

**Method:**

This retrospective study included 200 patients with T2DM who visited Prince Sattam Bin Abdulaziz University Hospital between January 2015 and December 2015. Venous blood was collected and examined for “serum/plasma levels of 25(OH)D” and related variables using kit methods. HbA1C levels <7% and ≥7% were taken as indicators of good and poor glycemic control, respectively. An association between vitamin D deficiency and poor glycemic control was determined using multinomial logistic regression analysis.

**Results:**

Among the total of 200 patients with type 2 diabetes, 118 (59%) were female and 82 (41%) were males with the mean age 42.4 ± 14.8 years. Good glycemic control (HbA1c < 7) was observed in 127 (63.5%), and poor glycemic control (HbA1c > 7) was found in 73(36.5%). The mean serum 25(OH)vit D was 20.27 ± 8.66 ng/mL, with (52% vs 82%; *P* ≤ .001) of subjects identified to have vitamin D deficiency in good and poor glycemic control groups, respectively.

**Conclusion:**

Taken together, our results demonstrated an association of vitamin D level with poor glycemic control in patients with type 2 diabetes. However, additional studies with larger sample size from local population are warranted in future to confirm and extend the findings of the present study.

## 1 INTRODUCTION

Diabetes is considered as a foremost public health epidemic worldwide that imposes a significant mortality and comorbidity attributable to macrovascular and microvascular complications.^1^ Effective diabetes therapies have been proposed during the past few years. While, novel insights to manage and prevent this condition remains necessary because of an amplified disease prevalence. Vitamin D has fascinated extensive interest in the past with respect to extra‐skeletal outcomes in several different disease conditions such as diabetes.^2^ Deficiency of vitamin D (ie also referred to as serum 25‐hydroxyvitamin D [25(OH)D] <50 nmol/L) is primarily prevalent in the diabetic population.^3^, ^4^, ^5^ In King Saud Bin Abdulaziz University, the diabetes mellitus prevalence is estimated to be 23.7%.^6^ World Health organization^7^ reported diabetes to be 8.3% among the age group of 20 and 70 years that was considerably expected to intensify to 10.1% in 2035. A contemporary review on different Saudi Arabia population by Al‐Daghri^8^ suggested that deficiency of vitamin D prevalence studied from 2011‐2016 was 81%.^8^ Glycated hemoglobin (HbA1c) is one of the markers to analyze the glycemic state of an individual over several years.^9^ At present, the levels of HbA1c are used to diagnose the diabetes and adjust therapies to manage diabetes.^10^ The diagnosis of diabetes by HbA1c levels is advantageous over fasting blood glucose or glucose tolerance test because its levels are least affected by the changes in glucose concentrations in response to illness or stress.^10^, ^11^

Previous evidences from cross‐sectional studies demonstrate an inverse relationship between hyperglycemia prevalence and vitamin D status.^4^, ^12^ Conversely, longitudinal studies show that low status of vitamin D is also a predictor for type 2 diabetes (T2DM) incidence.^13^, ^14^ Further clarity to whether vitamin D together with resistance from insulin is associated causally or whether they institute 2 autonomous features of patients with diabetes is needed. The findings from the previous interventional studies with the vitamin D supplementation have been contradictory. A meta‐analysis and systematic review of fifteen studies examining the effects of Vitamin D supplementation concludes that presently, there lies an inadequate evidence about the beneficial effect in regard to recommending the supplementation of vitamin D as a foundation to improve insulin resistance and glycemia in patients with diabetes, normal fasting glucose, and impaired glucose tolerance.^15^ A positive but weak vitamin D supplementation effect was observed on insulin resistance and fasting glucose in patients with diabetes. The inconsistency in these findings may be because several studies using a different supplementation regimen did not have glycemic control as a sole outcome and had lack of power or failed to include patients with diabetes. Overall, the causality of the relationship between glycemic control and vitamin D in patients with diabetes has not been confirmed.

The poor status of vitamin D may play a critical role in T2DM development. However, only some prospective studies on the association and mechanism exist and how vitamin D affects the T2DM risk is specifically not clear.^16^, ^17^, ^18^, ^19^ Therefore, it is essential to explore whether the vitamin D levels have any influence on glycemic control in Saudi patients with T2DM diabetes. The study aims to determine the frequency of good and poor glycemic control and an association between vitamin D deficiency in addition to poor glycemia control among patients presenting with T2DM.

## 2 METHODS

### 2.1 Study design and settings

A retrospective study was undertaken at Prince Sattam Bin Abdulaziz University Hospital located in the city of Al Kharj, Saudi Arabia, between January 2015 and December 2015.

### 2.2 Sampling technique/enrollment and patient selection

We used consecutive non‐probability sampling design for recruitment of the patients. Potential research subjects were determined from the list of patients with T2DM in Prince Sattam Bin Abdulaziz University Hospital at Al Kharj, based on the inclusion criteria. An appointment was scheduled before enrollment, where the patients were informed about the study and a written informed consent was obtained from all the patients. The status of vitamin D was established before including the patients in any group, to exclude patient who were vitamin D sufficient.

### 2.3 Sample size

Sample size of 200 cases is calculated with 95% confidence level, 7% margin of error together with capturing expected percentage of poor glycemia control, that is, 65.2% among patients with T2DM.

### 2.4 Data collection

The retrospective data were analyzed to identify patients having low vitamin D levels and those with poor and good glycemic control. Blood samples were obtained from all the patients for baseline investigation retrospectively. The samples were drawn at the same time and sent to the hospital laboratory for blood analysis. These routine laboratory test samples were processed immediately. The measurements were documented from the hospital records retrospectively. This included BMI, levels of vitamin D, fasting blood glucose, HbA1c, calcium, and creatinine levels in all patients. A Beckman Coulter AU analyzer was used to examine HbA1c.

### 2.5 Selection of participants (Criteria for inclusion and exclusion)

Inclusion criteria enclosed T2DM patients, non‐pregnant individuals, participants who did not receive any vitamin D supplementation during the past six months, and 18 years or older patients with diabetes who visited the outpatient clinics within the specified time. Exclusion criteria include pregnant females, hospitalized patients, or individuals having chronic diseases such as liver disease, uremia, cancer, lung disease or Cushing syndrome or using any steroid medicines.

### 2.6 Operational definition

The participants had diabetes if clinical history and fasting serum glucose concentration were > 126 mg/dL 19 mainly based on the guidelines of International diabetes federation. Participants who had vitamin D level less than 20 ng/mL were considered as having vitamin D deficiency. Participants were considered to have a glucose control if the HbA1c level was less than 7%.^1^

### 2.7 Data analysis

Data were evaluated using Statistical Package for Social Science (SPSS) software Version 23.0. Descriptive statistics that were obtained as frequencies and percentages were used for continuous variables. Independent sample t test was used for comparison. Chi‐square test was applied to examine the association between different categorical variables. *P*‐values < .05 were considered statistically significant. Multinomial logistic regression was used to estimate the strength of association between vitamin D deficiency and poor glycemic control. Data were stratified for the effect modifiers by using logistic regression.

### 2.8 Ethical approval

The ethical approval was attained from the “Institutional Review Board University Hospital of Prince Sattam Bin Abdulaziz University, Al Kharj, Saudi Arabia.” A written informed consent was also obtained from all the participants.

## 3 RESULTS

The subjects enrolled in the study comprised of 200 patients [118 female and 82 males] with type II diabetes having a mean age of (42.4 + 14.8 years). Around 82 patients were male while 118 were female. Table 1 represents the demographic and baseline laboratory characteristics of the patients. The mean BMI was 29.0 ± 6.5 kg/m^2^ with 32.5% patients being overweight and 42.5% obese. The findings of this study have also shown the mean HbA1c (%) level among all patients was 7.1 ± 1.98 of which good glycemic control (HbA1c < 7%) was observed in 111(56%) patients and poor glycemic control (HbA1c ≥ 7%) was apparent in 89 (44.5%). The mean of serum 25(OH) vitamin D was observed to be (20.27 ± 8.66 ng/mL) among all study participants.

**Table 1:** Baseline characteristics of the study participants

Characteristics	N (%)
Age in years (N = 200)	42.4 ± 14.8
(18‐40)	85 (42.5)
(41‐60)	94 (47.0)
(61‐90)	21 (10.5)
Gender (N = 200**)**	
Female	118 (59)
Male	82 (41)
BMI kg/m^2^ (N = 200)	29.0 ± 6.5
Underweight (<18 kg/m^2^)	7 (3.5)
Normal weight (18‐24.9 kg/m^2^)	43 (21.5)
Overweight (25‐29.9 kg/m^2^)	65 (32.5)
Obese (≥ 30 kg/m^2^)	85 (42.5)
Fasting Blood Glucose (mmol/L)	7.9 ± 3.7
(<7mmol/L)	116 (58)
(≥7mmol/L)	84 (42)
HbA1c (%)	7.1 ± 1.98
(<7)	127 (63.5)
(≥7)	73 (36.5)
Vitamin D level (ng/mL)	20.27 ± 8.66
Calcium (mmol/L)	2.3 ± 0.2
Creatinine (μmol/L)	62.2 ± 32.7

Note

Data are expressed as mean ± SD or frequency (%).

On comparison between the two groups of glycemic control, Table 2 described the insignificant differences for BMI, serum Ca++, and creatinine (*P*‐value > .05). However, the mean serum 25(OH) vitamin D was significantly higher in the control glycemic group (21.8 ± 8.9 vs 17.6 ± 7.5) as compared to study group with statistical difference *P* ≤ .001. Mean FBS were lower in the type II diabetes patients with their controlled glycemic levels being (5.9 ± 1.0 vs 11.3 ± 4.3) as compared to poor glycemic control group (≤0.001, Student's *t* test). Table 2 also demonstrated a significant inverse relationship between vitamin D deficiency and poor glycemic group. The frequency of vitamin D deficiency was 63%, and insufficiency was 23%. Results revealed that vitamin D deficiency was higher in poor glycemic group [82% vs 52%; *P* ≤ .001 (χ^2^ test)] as compared to controlled glycemic group.

**Table 2:** Comparison of biochemical and anthropometric variables according to glycemia control among patients with type II diabetes

	Poor glycemic control (n = 73)	Good glycemic control (n = 127)	*P*‐value
Male/female	42/31	40/87	<.05
Age (y)	45.3 ± 14.2	40.8 ± 14.9	<.05
BMI (kg/m^2^)	29.3 ± 6.4	28.9 ± 6.6	>.05
Serum Ca (mmol/L)	2.30 ± 0.1	2.26 ± 0.2	>.05
Serum creatinine	68.6 ± 51.3	58.4 ± 12.2	>.05
Fasting blood glucose(mg/dL)	11.3 ± 4.3	5.9 ± 1.0	≤.001
HbA1c (%)	9.1 ± 1.8	5.8 ± 0.5	≤.001
Serum 25(OH)vit D (ng/mL)	17.6 ± 7.5	21.8 ± 8.9	≤.001
Deficiency (<20)	66 (52)	60 (82)	≤.001
Insufficient (20.0‐29.9)	39 (31)	7 (10)	
Sufficient (> 30.0)	22 (17)	6 (8)	

Note

Data are presented as mean ± SD or frequency.

Chi‐square test and independent sample t test are used for comparison of categorical and continuous variables.

*P‐*value < .05 indicates a significant difference.

As illustrated in Table 3, multinomial logistic regression model analysis showed an association between the dependent variable, that is, 25(OH)D3 levels and the independent variable HbA1c (β: 0.300 [0.114‐0.790]) which shows a significant increase in odds (70%) of having uncontrolled diabetes existing in patients with a vitamin D deficiency, which is also adjusted for age, gender, and BMI.

**Table 3:** Association between vitamin D deficiency and poor glycemia control in patients with type II diabetes

Independent variables	Odds ratio (95% CI)	*P*‐value
Vitamin D (ng/mL)		
**<**20	0.300 [0.114‐0.790]	≤.001
20 to 29	1.519 [0.453‐5.091]	.782
≥30	1.00	Reference
Age (y)		
(16‐40)	1.966 [1.054‐3.669]	.037
(41‐60)	1.00	Reference
(61‐90)	1.257 [0.476‐3.318]	≤.001
Gender		
Male	0.339 [0.187‐0.616]	.200
Female	1.00	Reference
BMI (/kg/m^2^)		
Normal weight	1.00	Reference
Under weight	3.923 [0.433‐35.530]	.006
Over weight	1.370 [0.614‐3.056]	.517
Obese	0.981 [0.463‐2.076]	.020

Note

Multinomial logistic regression was used to evaluate the role of confounding variables for cases and controls.

## 4 DISCUSSION

The objective of this study was to determine the association between vitamin D deficiency and poor glycemic control among T2DM patients. Diabetes mellitus is increasing globally and has posed severe health and financial threats.^20^ In United States, the prevalence of diabetes from 1995 to 2004 increased from 14% to 23%.^21^ Saudi population tends to have high prevalence of type 2 DM and vitamin D deficiency. The incidence of diabetes increases with advancing age and has been reported between 22% and 33% in adults over 65 years. A further increase of 4.5 times has been projected by 2050 in US population.^22^ In Middle East, Saudi Arabia is ranked at number 2 and worldwide at number 7 in terms of diabetic population. Almost 7 million individuals are currently diagnosed with diabetes while more than 3 million are classified into pre‐diabetic category.^23^ It has also become a leading cause of morbidity among Saudi population.^20^

While comparing our results, mean age was found to be statistically significant with poor glycemic control (45.3 + 14.2) vs good glycemic control (40.8 + 14.9) and *P*‐value was less than and equal to .05. Therefore, patients having higher age had poor glycemic control. This makes it evident that glycemic levels tend to rise with the increasing age. Similarly, such trends were also observed in studies conducted by Hawthorne in 2011.^24^

Moreover, the females were found to have good glycemic control as compared with their male counterparts. The *P*‐value was found to be .05 which means both groups differ significantly with each other. While studies conducted in India showed male predominance in terms of poor glycemic control,^25^, ^26^ this is possible because of the fact that there were significantly more females than male diabetics enrolled in the current study.

Though mechanism of action is unclear, vitamin D is associated with the pathogenesis of T2DM. Vitamin D directly impacts the secretion, resistance, sensitivity, and action of insulin.^27^, ^28^, ^29^ Vitamin D through these functions helps in the regulation of glycemic state of the body (HbA1c). There are many potential mechanisms concerning vitamin D affecting glycemic control in diabetic population. Majority of the cells, such as the pancreatic β‐cells, comprise of vitamin D receptors while majority owns a capacity to yield biologically active 1,25‐dihydroxyvitamin D [1,25(OH)2D], that permits paracrine in addition to intracrine functions. In vitro studies demonstrate an active vitamin D metabolite 1,25(OH)2D stimulated insulin release by the pancreatic β‐cells.^30^ Moreover, vitamin D tends to have anti‐inflammatory and immunomodulatory effects that may limit peripheral insulin resistance by changing low‐grade chronic inflammation.^10^, ^11^ Insulin sensitivity together with insulin secretion is equally considered to be calcium‐dependent processes. Several cross‐sectional studies have suggested an inverse association between blood vitamin D levels along with HbA1c not only in patients but also in normal adults.^31^, ^32^, ^33^, ^34^, ^35^

Vitamin D deficiency has been reported worldwide and has affected around one billion population.^36^ Its deficiency has been considered a potential risk factor for death in patients suffering from cardiovascular diseases and cancers.^36^ Therefore, it is of great value to diagnose the deficiency at earlier stage and look for optimal remedial measures to avoid such complications. In Kingdom of Saudi Arabia, vitamin D deficiency is at rise along with increased incidence of diabetes. For example, in western region, 80% of the female population is reported to be vitamin D‐deficient.^16^ Similar results were found in eastern region of the country where even population was having sufficient dairy products and sun exposure.^37^ In this context, the primary goal of this study was to measure and then correlate the levels of vitamin D with HbA1c in patients with diabetes. Despite a year‐long plenty of sunlight in different countries for instance, Asia and Middle East, deficiency of vitamin D^38^, ^39^ is common among diverse populations in different age‐groups.^23^, ^40^, ^41^, ^42^, ^43^, ^44^, ^45^, ^46^, ^47^ This was specifically observed in China, Thailand, Lebanon, Morocco, Jordan, Saudi Arabia, and Indian sub‐continent population. Poor dietary intake and sunlight avoidance especially among Saudi nationals are possibly the key risk factors for the deficiency of vitamin D. According to Darraj,^48^ in Jazan City, Saudi Arabia, vitamin D deficiency (VDD) is highly prevalent in individuals with T2DM and is associated with poor glycemic control.

Our results showed that type 2 diabetic population had deficiency of vitamin D. The mean HbA1c (%) level among all patients was 7.06 + 1.98 of which good glycemic control (HbA1c < 7%) was observed in 111 (55.5%) and poor glycemic control (HbA1c > 7%) was apparent in 89 (44.5%). Mean values of serum 25(OH) vitamin D were discernibly lower and HbA1c was higher than the respective reference values used in our laboratory. These findings pose serious questions regarding the well‐being of the Saudi populations. As the consequences of low vitamin D have been allied with progression of diabetes and poor glycemic control, being consistent with the results obtained in this study. This is evident in the study conducted by Kaya et al^47^that demonstrates that vitamin D deficiency was related to poor glycemic control in patients with diabetes.

The risk of lower vitamin D is considerably higher in Saudi population because of the climate and cultural reasons. For example, only 4.6% young school students (6‐19 years) showed adequate levels of vitamin D, whereas 95.4% had deficiency. Likewise, when adults were screened (20‐62 years), 89.1% were found deficient.^38^ This high percentage of population warrants attention on this issue and demands formulation of guidelines for proper dosage and duration of vitamin D supplementation. The literature supports the idea that even in healthy non‐diabetic individuals’ proper administration of vitamin D may help in lowering the HbA1c levels.^17^ In a recent study, vitamin D supplementation has shown a decline in HbA1c levels from 5.6% to 5.5% in non‐diabetic patients with above average vitamin D levels and those who were below baseline levels but improved after vitamin D intake. Although a decline of 0.1% seems very small, lowering of HbA1c levels from 0.1% to 0.2% have shown to reduce the risk of diabetes development by 6% and 13%, respectively, in self‐reporting patients with diabetes.^18^ Further, 0.007% decrease in HbA1c levels have been reported with every 25 nmol/L increase in vitamin D levels.^49^

Despite several studies 47, 50 have established an inverse relationship between vitamin D and HbA1c levels, others fail to demonstrate such association.^16^ Direct association between vitamin D and HbA1c levels was not established but stimulated insulin secretion was reported because of vitamin D supplementation. Mechanism through which vitamin D can influence metabolism of glucose in patients with diabetes comprises of improvement in insulin sensitivity and β‐cell survival, amplified secretion of insulin from pancreatic β‐cells, calcium flux regulation for normalizing glucose tolerance, and β‐cell protection against cytokine‐induced apoptosis.^4^ Another study conducted by Ahmadieh et al in 136 diabetic patients demonstrated that serum 25‐OHD is negatively correlated with HbA1c.^51^

The findings from the previous studies focusing on the treatment of vitamin D through supplementation have been conflicting. A meta‐analysis/systematic review of fifteen research studies examined the impact of vitamin D supplementation. The results revealed inadequate evidence of useful effect to recommend vitamin D supplementation to improve glycemia in patients with normal fasting glucose, impaired glucose tolerance, or diabetes.^52^ However, a weak positive effect was also observed with vitamin D supplementation on fasting glucose in T2DM patients. This inconsistency was because several studies included used different regimens for supplementation, did not use glycemic control as a primary outcome, and had lack of power. Conversely, in other studies, supplementation of vitamin D had improved glucose control in patients with T2DM.^53^, ^54^

Our study had certain limitations. The study design made it hard to create a causal association between HbA1c levels and vitamin D. Moreover, the investigation is derived from solely a single HbA1c measurement and vitamin D levels. The study was also carried out at Prince Sattam Bin Abdulaziz University Hospital using consecutive non‐probability sampling design for recruitment of the patient and results cannot be generalized to the entire Saudi population. Despite these limitations, our study provided evidence pertaining to the role of vitamin D in glycemic control in Saudi patients with diabetes. Nevertheless, our observations have opened future area of discussion and research which requires systemic approach to measure the levels of vitamin D and HbA1c not only in patients with diabetes but also in non‐diabetics on a larger scale. This will allow scientific community to devise suitable guidelines for vitamin D therapy and management of diabetes.

## 5 CONCLUSION

We conclude that the poor status of vitamin D may play a critical role in T2DM development. The population in Saudi Arabia is generally insufficient in 25OH vitamin D. We found an association of vitamin D level with poor glycemic control in patients with type 2 diabetes. This association points toward the role of vitamin D supplementation along with raising awareness about hypovitaminosis D in the diabetic population that could possibly play a significant role in glycemic regulation in these patients.

## 6 CLINICAL IMPLICATIONS AND RECOMMENDATION FOR FUTURE RESEARCH

Based on the results generated from our study, patients with T2DM having poor glycemic control should be screened regularly for deficiency of vitamin D in order to limit the burden of morbidity and mortality in Saudi Arabia. Patients with T2DM also having vitamin D deficiency should also be treated with vitamin D supplementation to improve indirect health‐related outcomes. Further studies with larger sample size from local population as well as those on vitamin D supplementation in T2DM patients are needed in future, to confirm and extend the findings of the current study.

## AUTHOR CONTRIBUTIONS

KKA designed the study. GA, AA, NA, MBS, MA, DA, FBH, and AAQ performed data acquisition. KKA and SA performed the data analysis, and HAR drafted the study. All authors read and approved the final study for submission.

## ETHICAL APPROVAL

The study was approved by the “Institutional Review Board University Hospital of Prince Sattam Bin Abdulaziz University, Al Kharj, Saudi Arabia.” All analytical tests/procedures performed in this study were in accordance with the ethical standards of the institutional research committee and in accordance with the 1964 Helsinki declaration and its amendments. Informed consent was obtained from all individual participants included in the study.”

## Data Availability

The datasets used and analyzed during the current study are available from the corresponding author on reasonable request.
