# Corrigendum

**DOI:** 10.1002/jcla.23591

**Published:** 2020-10-29

In Volume 34, Issue 2 (2019),^1^ in the article titled “Association of vitamin D with glycemic control in Saudi patients with type 2 diabetes: A retrospective chart review study in an emerging university hospital” by Khaled K. Al Dossari, Gulfam Ahmad, Abdulrahman Aljowair, Naif Alqahtani, Mohammed Bin Shibrayn, Mohammed Alshathri, Dahfer Alshehri, Suhair Akhlaq, Faisal Bin Hejab, Abdulelah Alqahtani, Hira Abdul Razzak, the author missed adding the following lines to the Acknowledgments.

This publication was supported by the Deanship of Scientific Research at Prince Sattam bin Abdulaziz University, Al Kharj, KSA. We are thankful to the deanship for this support.
